# Automated EEG-based sleep staging in REM sleep behavior disorder using MSIF-Net: epoch-level validation and whole-night clinical agreement

**DOI:** 10.3389/fneur.2026.1819351

**Published:** 2026-05-19

**Authors:** Huanyu Li, Tianxing Li, Mengxue Wang, Alan Luiz Eckeli, Yuan Zhang, Zhiyuan Sun, Yuanyuan Che, Yudan Lv

**Affiliations:** 1 Neuroscience Center, Department of Neurology, The First Hospital of Jilin University, Changchun, China; 2 Key Laboratory of Bionic Engineering, Ministry of Education, Jilin University, Changchun, China; 3 Department of Neurosciences and Behavioral Sciences-FMRP, Universidade de São Paulo, São Paulo, Brazil; 4 College of Electronic and Information Engineering, Southwest University, Chongqing, China; 5 Changchun Institute of Optics, Fine Mechanics and Physics, Chinese Academy of Science, Changchun, Jilin, China; 6 Clinical Laboratory, The First Hospital of Jilin University, Changchun, China

**Keywords:** automated sleep staging, deep learning, EEG, REM sleep behavior disorder (RBD), sleep architecture

## Abstract

**Objective:**

REM-stage abnormalities are central to the pathophysiology of REM sleep behavior disorder (RBD) and are clinically relevant to symptom burden. We developed an EEG-based, pathology-oriented automated sleep-staging framework for RBD and tested whether model-derived REM architecture metrics are reliable and clinically informative in real-world polysomnography (PSG).

**Methods:**

The Multi-Stream Imaging Fusion Network (MSIF-Net) integrates raw EEG waveforms (1D CNN), time–frequency spectrograms (2D CNN), and 65 handcrafted descriptors via attention-based fusion. Stage 1 used patient-wise five-fold cross-validation for epoch-level Wake/NREM/REM staging. Stage 2 applied the fixed model to an independent clinical RBD cohort to estimate whole-night NREM% and REM% (of TST), evaluate agreement with routine manual summaries (Bland–Altman), and test associations between automated REM% and symptom scales (Spearman, FDR-corrected).

**Results:**

MSIF-Net achieved class-wise F1 scores of 0.84 for Wake, 0.94 for NREM, and 0.80 for REM, with errors mainly reflecting REM–wake confusions. Whole-night REM% and NREM% showed close agreement with manual summaries. Automated REM% correlated inversely with PSQI (*ρ* = −0.507, *q* < 0.001) and RBDSQ (*ρ* = −0.454, *q* = 0.002), but not ESS (*ρ* = −0.102, *q* = 0.667).

**Conclusion:**

MSIF-Net enables EEG-only three-class sleep staging in RBD and yields clinically consistent whole-night REM architecture estimates that capture clinically meaningful variation in sleep complaints and RBD symptom burden.

## 1 Introduction

Sleep is a vital physiological process comprising two major states: non-rapid eye movement (NREM) sleep and rapid eye movement (REM) sleep, each associated with characteristic brain activity, physiological regulation, and behavioral patterns (1–3). Disruptions in the architecture of these states are increasingly recognized as early markers of neurodegenerative disorders, particularly synucleinopathies such as Parkinson’s disease (PD) and REM sleep behavior disorder (RBD) (4–6). In particular, RBD, defined by the loss of normal REM atonia and the presence of dream-enactment behaviors, is regarded as one of the most specific prodromal markers of PD and other *α*-synucleinopathies (7–11). Given its predictive value, accurate characterization of sleep stage dynamics, especially REM-related alterations, is critical for advancing the early detection, pathophysiological understanding, and individualized management of these disorders (12–15).

Traditionally, assessment of RBD has relied on subjective questionnaires together with manual polysomnography (PSG), whereas definitive diagnosis still requires video-PSG with evidence of REM sleep without atonia and/or REM-related motor events (16). Although manual PSG remains the clinical reference standard, it is labor-intensive, time-consuming, and subject to inter-rater variability, which limits scalability for longitudinal monitoring and large-cohort studies (17, 18). In response, automated sleep staging based on EEG and multimodal PSG has advanced rapidly in recent years, evolving from conventional feature-engineering pipelines to convolutional, recurrent, and Transformer-based deep-learning frameworks (19–24). Recent studies suggest that these approaches have achieved promising performance in general sleep-staging settings and are increasingly being considered for clinical translation (25, 26). However, most mature models have been developed in healthy or non-neurodegenerative populations rather than in disorder-specific cohorts with pathological REM physiology (27–29).

In contrast, evidence in RBD remains limited and consistently indicates that automated stage recognition becomes more difficult under pathological REM conditions (30, 31). In a fully automated PSG pipeline, Cooray et al. (32) reported substantially lower sleep-staging agreement in RBD than in healthy controls (*κ* = 0.54 vs. 0.73), with REM sensitivity reduced to 0.45 ± 0.30 and frequent misclassification of REM epochs as wake. More recently, van der Aar et al. showed that both ExG-based and HRV/movement-based automated sleep staging remain less accurate in RBD than in a matched comparison group, and further demonstrated that disagreement is concentrated in REM and light NREM stages, where stage probabilities become more ambiguous (33). Likewise, Jung et al. found that a pretrained U-Sleep–based REM detector performed significantly worse in RBD than in non-RBD cohorts (AUC 0.88 ± 0.13 vs. 0.93 ± 0.14), with the lowest performance observed in PD with RBD (AUC 0.86 ± 0.02) (34). Together, these findings indicate that atypical REM physiology in RBD or PD is not merely a nuisance factor, but a recurrent source of REM–wake and stage-boundary ambiguity that can degrade the robustness of automated sleep staging. This highlights the need for sleep-staging models that are explicitly evaluated under pathological REM conditions in clinically acquired PSG, and optimized for robust, stage-resolved characterization of REM-related abnormalities.

Recent RBD-oriented digital studies also suggest that the field is expanding in complementary directions. Chen et al. (35) proposed a multi-task framework that jointly performs sleep staging and REM sleep without atonia (RSWA) detection, highlighting growing interest in integrated RBD analysis rather than isolated staging alone. In parallel, home-based digital screening approaches are emerging: for example, Tzfoni et al. (36) recently reported that a lower-back wearable sensor could identify iRBD-associated nocturnal motor patterns over multiple nights at home with high sensitivity but only moderate specificity, supporting its potential role in staged screening or cohort enrichment, rather than as a replacement for stage-resolved EEG characterization of sleep architecture. These advances are important and clinically relevant, but they also leave open a related question that remains insufficiently addressed in clinically acquired RBD PSG: whether pathology-aware EEG-based sleep staging can yield clinically interpretable whole-night sleep-architecture estimates. This issue is especially challenging in RBD because disorder-specific features can blur canonical distinctions between sleep stages.

More specifically, in patients with RBD, the accuracy of automated PSG-based sleep staging is challenged by several disorder-specific factors that alter physiological signal patterns and complicate conventional stage boundaries. First, RBD patients often show atypical transition dynamics between NREM and REM sleep, with less distinct electrophysiological signatures at stage boundaries, which complicates both manual and automated classification (33, 37). Second, the hallmark feature of RBD—loss of REM atonia—introduces EMG activity into REM periods, disrupting the canonical criterion used in both visual and automated scoring, thereby increasing misclassification rates (33, 34, 38). Third, comorbid conditions such as Parkinson’s disease and the chronic use of dopaminergic agents, antidepressants, or hypnotics further alter normal sleep architecture, adding variability that challenges robust staging in clinical populations (39–41). In addition, RBD sleep is frequently fragmented, with brief arousals and rapid transitions. Within a 30-s epoch of PSG, mixed-state segments can occur, increasing label ambiguity even for expert scorers. Collectively, these disorder-specific alterations highlight the need for staging algorithms capable of pathology-aware representation learning and adaptive robustness strategies to achieve reliable performance in RBD-related pathological sleep (42).

To address these challenges, we developed MSIF-Net, an EEG-based automated sleep-staging framework tailored to RBD. MSIF-Net integrates three complementary EEG representations within a unified multi-stream architecture: raw time-domain waveforms, time–frequency spectrograms, and physiologically interpretable handcrafted features. An attention-based fusion module adaptively reweights these representations at the epoch level to improve robustness when individual representations are degraded by artifacts or inter-individual variability, both common in clinical RBD recordings. Moreover, we evaluated MSIF-Net in a 2-stage design. In Stage 1 (technical validation), we assessed epoch-level performance using patient-wise five-fold cross-validation. In Stage 2 (clinical translation), we applied the fixed model to an independent clinical RBD cohort and evaluated agreement between automated and routine manual summaries for whole-night NREM% and REM% expressed relative to total sleep time (TST), and examined associations between model-derived REM% and symptom scales.

This study makes three primary contributions. First, we introduce a pathology-oriented, multi-representation EEG framework for automated sleep staging in RBD, designed to address the REM–wake ambiguity that commonly arises under atypical REM physiology. Second, rather than focusing primarily on performance in generic sleep cohorts, we evaluate the framework directly in clinically acquired RBD PSG and frame its output in terms of clinically interpretable whole-night REM/NREM sleep-architecture estimation. Third, we demonstrate clinical translation by showing that automated whole-night REM% and NREM% (of TST) closely agree with routine manual PSG summaries and that automated REM% captures clinically relevant variation in sleep quality and RBD symptom burden. Together, this work supports EEG-based automated staging as a scalable approach for REM-focused sleep-architecture characterization in RBD.

## 2 Methods

### 2.1 Study design

**Figure 1:**
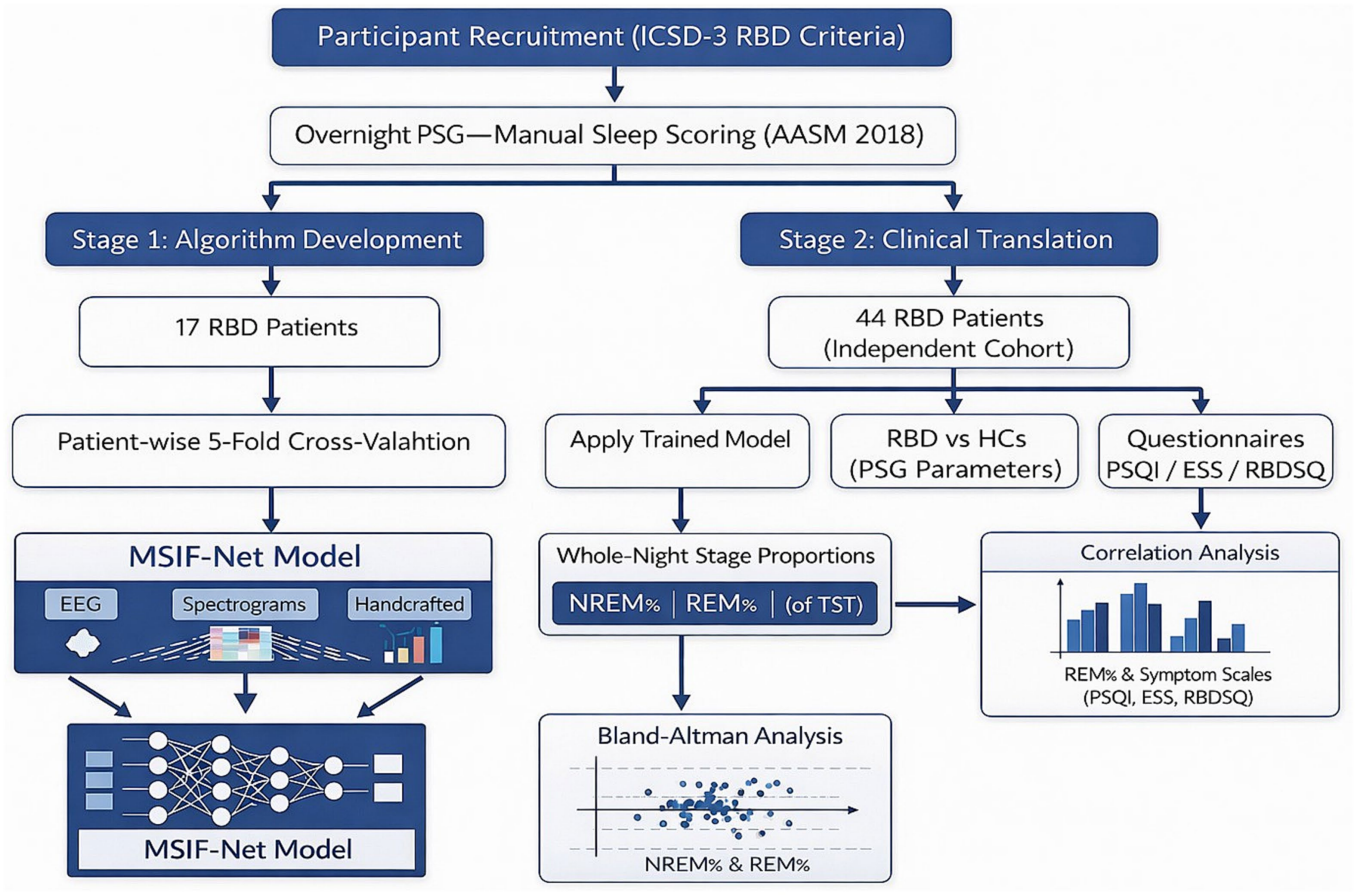
Overall study workflow. The study followed a two-stage, two-cohort design. Stage 1 developed and technically validated MSIF-Net using PSG-derived EEG data from 17 patients with RBD with adjudicated sleep-stage labels. Stage 2 assessed clinical translation in an independent, non-overlapping RBD cohort (n = 44) by deriving whole-night REM% and NREM% from automated staging and evaluating agreement with routine manual summaries, as well as associations with symptom scales (PSQI, ESS, RBDSQ). An additional 44 healthy controls were included only for group-level comparisons of clinical and PSG parameters with the Stage 2 RBD cohort and were not used for model development or agreement analyses.

The study followed a two-stage, two-cohort design (Figure 1). Stage 1 (algorithm development and technical validation) was used to develop and technically validate the Multi-Stream Imaging Fusion Network (MSIF-Net) using PSG recordings from 17 patients with RBD. These recordings contained adjudicated sleep-stage labels and were used exclusively for model development and technical evaluation. Stage 2 (clinical translation) applied the trained model (fixed after Stage 1) to an independent, non-overlapping RBD cohort (n = 44) with full-night PSG to derive participant-level estimates of NREM% and REM% and to evaluate agreement with routine manual summaries under real-world clinical conditions. In addition, 44 age- and sex-matched healthy controls (HCs) were recruited for group-level clinical and polysomnographic comparisons with the Stage 2 RBD cohort. HCs data were not used for model development, technical validation, or automated-manual agreement analyses. Because Stage 1 and Stage 2 served different purposes and differed in data completeness, analyses were prespecified separately: Stage 1 focused on technical model performance, whereas Stage 2 focused on clinical translation and symptom-association analyses.

### 2.2 Participants

Patients with clinically suspected RBD were consecutively recruited from the Sleep Disorders Clinic of the First Hospital of Jilin University between January and June 2025. The diagnosis of RBD was established according to the standardized criteria of the International Classification of Sleep Disorders, Third Edition (ICSD-3). All RBD participants underwent overnight laboratory-based polysomnography (PSG) as part of their diagnostic evaluation. Exclusion criteria for RBD participants included other major neurological disorders and major psychiatric disorders that could confound sleep architecture, including clinician-diagnosed schizophrenia spectrum or other psychotic disorders, bipolar disorder, and current severe major depressive disorder (e.g., requiring hospitalization or intensive treatment), as well as other psychiatric conditions associated with substantial functional impairment that could confound sleep architecture.

Stage 1 included 17 patients with RBD whose PSG recordings contained adjudicated sleep-stage labels suitable for algorithm development and technical validation. Because this cohort was used specifically for model development, only PSG-derived EEG signals and reference sleep-stage labels were used in the present study. Standardized symptom questionnaires were not available for all Stage 1 participants and therefore were not included in clinical association analyses.

Stage 2 included an independent, non-overlapping cohort of 44 patients with RBD who underwent full-night PSG and completed standardized clinical questionnaires, including the Pittsburgh Sleep Quality Index (PSQI), the Epworth Sleepiness Scale (ESS), and the RBD Screening Questionnaire (RBDSQ). These data were used for clinical translation analyses, including automated-manual agreement on whole-night sleep-stage proportions and associations between model-derived REM% and symptom burden. In parallel, 44 age- and sex-matched HCs were recruited during the same period for group comparisons of PSG-derived sleep characteristics. HCs had no history of neurological or psychiatric disorders, systemic disease, or moderate-to-severe sleep disorders, as confirmed by clinical history, questionnaires, and PSG screening.

### 2.3 Polysomnography acquisition and scoring

Overnight PSG was performed using a standard digital system (Kangdi, model Grael). EEG, electro-oculogram (EOG), and submental and anterior tibialis electromyogram (EMG) channels were recorded to characterize sleep stages and muscle activity. Respiratory signals included nasal airflow, thoracic and abdominal respiratory effort, oxygen saturation (SpO₂), and snoring detection. All recordings were conducted under standard conditions in the sleep laboratory. Sleep stages and respiratory events were independently and randomly scored by two registered polysomnographic technologists (RPSGT) according to the 2018 guidelines of the American Academy of Sleep Medicine (AASM). Discrepancies between the two RPSGT scorers were adjudicated by a third specialist, and the adjudicated labels were used as the reference standard for analysis.

### 2.4 Data preprocessing

We selected eight routinely available PSG-derived EEG electrodes (Fp1, Fp2, C3, C4, O1, O2, A1, and A2) together with their corresponding sleep-stage labels (0: wakefulness, 1: non–rapid eye movement [NREM] sleep, 2: rapid eye movement [REM] sleep). These channels were chosen *a priori* because they reflect a standard clinical PSG-derived EEG signals configuration and provide broad anterior–central–posterior coverage of sleep-related electrophysiological patterns. Specifically, frontal leads (Fp1/Fp2) capture low-frequency and transition-related activity, central leads (C3/C4) are informative for canonical NREM features such as K-complexes and sleep spindles, and occipital leads (O1/O2) help characterize posterior alpha activity relevant to wakefulness. Bilateral mastoid channels (A1/A2) were retained to preserve the routinely recorded PSG montage and its spatial information. Although EOG and EMG were available during standard PSG acquisition and contributed to manual reference scoring, they were not included as model inputs because the aim of the present study was to evaluate EEG-based sleep staging performance in isolation rather than to develop a multimodal PSG staging system or to automate RBD diagnosis or RSWA detection. Continuous EEG signals were first band-pass filtered between 0.5 and 35 Hz to remove baseline drift and high-frequency noise. Moreover, we applied discrete wavelet transform (DWT)–based denoising to suppress transient, nonstationary noise while preserving sleep-related EEG morphology. To ensure data quality, abnormal segments were automatically detected using statistical criteria (eg, signal amplitude, variance, and spectral features), and artifacts were either removed or corrected. The preprocessed EEG signals were segmented into 30-s epochs, from which multiple time- and frequency-domain features were extracted, yielding a 65-dimensional feature vector for each epoch. These feature vectors were used for subsequent statistical and machine learning analyses.

### 2.5 Model architecture (MSIF-Net)

**Figure 2:**
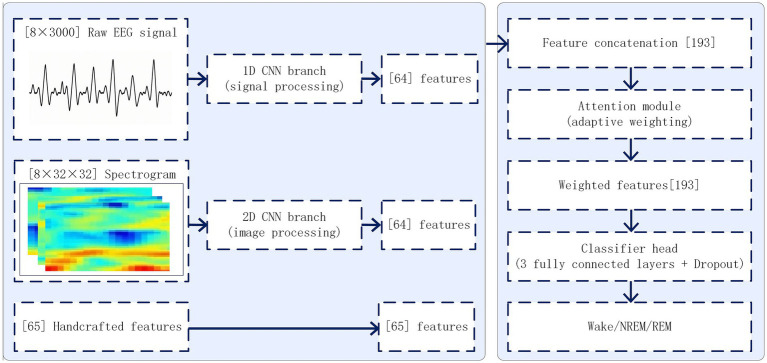
Overview of the proposed Multi-Stream Imaging Fusion Network (MSIF-Net). The model integrates three parallel input streams: raw EEG signals processed by a 1D CNN branch, time–frequency spectrograms processed by a 2D CNN branch, and handcrafted EEG features. Features from the three streams are combined through an attention-based fusion module, which adaptively weights each representation to enhance complementary information. The fused features are then passed to a fully connected classifier consisting of three dense layers with dropout regularization, yielding the final sleep stage probabilities (Wake, NREM, REM).

To comprehensively capture the complementary information of EEG signals, we designed a multi-stream feature integration network (MSIF-Net) consisting of three parallel branches (Figure 2). The detailed layer configuration of MSIF-Net is provided in Supplementary material S1.

#### 2.5.1 Signal processing branch (1D CNN stream)

Each 30-s epoch was represented as an eight-channel raw EEG segment (100 Hz, 3,000 samples/channel). The 1D stream consisted of three convolutional blocks (convolution–normalization–activation–pooling) followed by global aggregation to yield a fixed-length 64-dimensional time-domain embedding (full layer configuration in Supplementary material S1). This branch captures short-range temporal morphology and dynamics directly from raw waveforms.

#### 2.5.2 Spectrogram processing branch (2D CNN stream)

In parallel, each 30-s EEG epoch was transformed into a channel-wise time–frequency representation (spectrogram; computed using STFT, details in Supplementary material S1), resized to a 32 × 32 grid, and stacked across the 8 channels as a multi-channel 2D tensor input. A 2D CNN was then used to learn spectro-temporal patterns and cross-channel dependencies associated with canonical sleep oscillations (e.g., spindles and slow-wave activity).

#### 2.5.3 Handcrafted feature branch

In parallel with the deep-learning streams, we computed a 65-dimensional handcrafted feature vector for each 30-s EEG epoch (Supplementary material S2). The handcrafted features summarize clinically interpretable EEG characteristics, including time-domain amplitude and variability statistics (eg, mean, standard deviation, variance, and root mean square), frequency-domain band-limited power in canonical bands (delta, theta, alpha, beta, and low-gamma [30–35 Hz]) estimated using Welch’s method, and spectral edge frequency (SEF90). These features were selected to encode established sleep-physiology priors and to provide an interpretable, complementary representation to CNN-derived embeddings, which may be advantageous when training data are modest and signal quality varies across clinical PSG recordings.

#### 2.5.4 Feature fusion and classifier

The three types of features—temporal (1D CNN), spectro-temporal (2D CNN), and handcrafted were concatenated into a 193-dimensional feature vector and integrated using a lightweight feature-wise gating MLP (two-layer MLP with sigmoid gating; details in Supplementary material S1). The gated features were then fed into a fully connected classifier with dropout regularization to output probabilities for Wake, NREM, and REM.

#### 2.5.5 Model training and cross-validation

Sleep stage classification was performed at the 30-s epoch level. Stage 1 technical validation was conducted on PSG recordings from 17 patients with RBD using patient-wise five-fold cross-validation, such that all epochs from a given participant were assigned to the same fold to prevent information leakage. In each iteration, data from four folds were used for model development, and the remaining fold served as the held-out test set. Within the development folds, a subset of participants was further separated as an internal validation set for early stopping and model selection. Accordingly, model training and internal validation were performed only within the development folds, whereas performance evaluation was performed exclusively on the held-out test fold, which was never used for model fitting, early stopping, or model selection. The model was optimized using the Adam optimizer with an initial learning rate of 0.001. To address class imbalance, we used weighted cross-entropy loss, and a learning-rate scheduler was applied according to validation performance. For each cross-validation iteration, the model state with the best internal validation performance was retained and then used to generate predictions for the held-out test fold. Final epoch-level performance was summarized by pooling predictions from the held-out test fold across all five cross-validation iterations, ensuring that each epoch was evaluated only when its source participant had not been used for model training.

For stage-wise visualization only, we additionally drew a class-balanced subset of held-out epochs (*N* = 1,389; 463 per class) from the pooled held-out predictions to display the confusion matrix and facilitate visual comparison across sleep stages. This visualization subset was not used for model training, model selection, or primary performance evaluation. Its purpose was solely to avoid visual dominance by the majority class; accordingly, the corresponding accuracy should be interpreted only as a class-balanced visualization estimate rather than as reflecting the natural epoch distribution of overnight PSG.

### 2.6 Clinical application of MSIF-Net and sleep characteristics in RBD

After cross-validation-based model selection in Stage 1, we retrained a final MSIF-Net on the full Stage 1 cohort using the fixed preprocessing pipeline, network architecture, class weights, and optimization settings. This finalized model was then frozen and applied to the independent, non-overlapping Stage 2 RBD cohort (*n* = 44), in which the clinical translation of MSIF-Net was evaluated using full-night PSG. For each recording, the frozen model generated automated 30-s sleep-stage labels, from which participant-level proportions of NREM sleep, and REM sleep were derived. Given the large number of epochs per participant and the strong temporal autocorrelation among consecutive epochs within the same individual, Stage 2 validation focused on whole-night stage proportions rather than additional epoch-level agreement analyses. Because REM% and NREM% were expressed relative to TST, the night-level agreement analyses were designed to assess sleep-stage composition within sleep rather than comprehensive wake-related staging performance. Agreement between automated and routine manual summaries was therefore evaluated for REM% and NREM% of TST using Bland–Altman analyses. Because REM sleep disruption is a characteristic feature of RBD, we further examined the clinical relevance of automated staging by testing associations between model-derived REM% and symptom scales (PSQI, ESS, and RBDSQ). In parallel, conventional PSG-derived sleep parameters were compared between the Stage 2 RBD cohort and age- and sex-matched healthy controls. Within the RBD cohort, standard PSG measures (e.g., sleep efficiency, sleep latency, and arousal index) were also examined in relation to the same clinical scales to further contextualize the clinical significance of altered sleep architecture.

### 2.7 Statistical analysis

Data were first examined for normality using the Kolmogorov–Smirnov test and for homogeneity of variance using Levene’s test. Continuous variables are presented as mean ± SD when normally distributed and as median (IQR) otherwise; categorical variables are summarized as number (percentage). For group comparisons between patients with RBD and HCs, normally distributed variables with equal variances were analyzed using independent-samples t tests; otherwise, the Mann–Whitney U test was applied. Categorical variables were compared using *χ*^2^ tests. Agreement between manual and automated scoring of sleep stage proportions was assessed using Bland–Altman analyses. In addition, mean absolute error (MAE) was calculated as the mean of the absolute differences between automated and manual stage proportions across participants. Intraclass correlation coefficients (ICC) were computed using a two-way mixed-effects model with absolute agreement (single measurement) to quantify agreement between automated and manual estimates, and 95% confidence intervals were calculated. Within the RBD cohort, associations between automated REM proportion and manual-derived PSG sleep parameters with clinical scales (PSQI, ESS, and RBDSQ) were examined using Spearman’s correlation coefficients.

To further evaluate independent associations, multivariable linear regression models were constructed with each clinical scale as the dependent variable and automated REM% or other relevant PSG parameters as predictors, adjusting for demographic and clinical covariates (age, sex, BMI, and medication use when available). All tests were 2-tailed, and a *p* value < 0.05 was considered statistically significant.

## 3 Results

### 3.1 Demographic and clinical characteristics

A total of 44 patients with RBD and 44 age- and sex-matched HCs were included in the final Stage 2 analysis (Table 1). The two groups did not differ significantly in age, sex distribution, or BMI (all *p* > 0.05). As expected, the RBD group had markedly higher scores on the PSQI, ESS and RBDSQ compared with HCs (*p* < 0.001).

**Table 1:** Demographic and clinical characteristics of patients with RBD and healthy controls.

Variables	RBD (*n* = 44)	HCs (*n* = 44)	*p*-value
Age, years	64.82 ± 8.94	63.66 ± 7.92	0.406
Male/Female, *N*	27/17	28/16	0.828
BMI, kg/m^2^	23.39 ± 3.08	23.60 ± 2.78	0.679
Medication use, *n* (%)	13 (29.55)	—	—
Antidepressants (SSRI/SNRI/TCA), *n* (%)	5 (11.36)	—	—
Benzodiazepines or Z-drugs, *n* (%)	3 (6.82)	—	—
Melatonin or melatonin receptor agonists, *n* (%)	3 (6.82)	—	—
Other sedatives/anxiolytics, *n* (%)	2 (4.55)	—	—
PSQI	7.00 (6.75–8.00)	5.00 (4.00–5.00)	<0.001
ESS	7.00 (6.00–7.25)	4.00 (3.00–5.00)	<0.001
RBDSQ	7.25 (6.50–8.00)	—	—

Continuous variables are presented as mean ± SD for normally distributed data and as median (IQR) for non-normally distributed data; categorical variables are presented as *n* (%). Any sleep- or REM-modulating medication was defined as current use of antidepressants (SSRI/SNRI/TCA), benzodiazepines or Z-drugs, melatonin or melatonin receptor agonists, or other sedatives/anxiolytics. Abbreviations: BMI, body mass index; ESS, Epworth Sleepiness Scale; HCs, healthy controls; PSQI, Pittsburgh Sleep Quality Index; RBD, REM sleep behavior disorder; RBDSQ, REM Sleep Behavior Disorder Screening Questionnaire; REM, rapid eye movement; SNRI, serotonin–norepinephrine reuptake inhibitor; SSRI, selective serotonin reuptake inhibitor; TCA, tricyclic antidepressant.

Compared with HCs, patients with RBD exhibited significantly shorter total sleep time and lower sleep efficiency (both *p* < 0.001) (Table 2). Sleep architecture was notably altered, with increased N1 and reduced N2, N3, and REM proportions (all *p* < 0.05). The arousal index and Limb movements during REM sleep (R-LM) burden were also elevated in RBD (all *p* < 0.01), reflecting greater sleep fragmentation and motor activity during REM sleep. These findings confirm the characteristic sleep disturbances in RBD and provide a quantitative basis for subsequent algorithm-based analyses.

**Table 2:** Polysomnographic parameters in RBD and healthy control groups.

Polysomnographic parameter	RBD (*n* = 44)	HCs (*n* = 44)	*p*-value
Time in bed (min)	524.55 ± 48.29	516.20 ± 41.39	0.200
Total sleep time (min)	335.01 ± 67.06	408.44 ± 41.93	<0.001
Sleep efficiency (%)	63.12 (53.59–72.70)	78.36 (74.73–83.98)	<0.001
Sleep latency (min)	24.00 (19.13–28.88)	19.00 (16.13–26.75)	0.011
Arousal Index (events/h)	20.92 ± 8.47	15.38 ± 5.45	0.003
N1 (% TST)	33.90 ± 13.09	20.77 ± 7.13	<0.001
N2 (%TST)	48.30 ± 13.36	55.41 ± 6.90	0.006
N3 (%TST)	2.10 (0.40–6.90)	5.90 (2.05–10.30)	0.015
REM (%TST)	12.65 (7.73–18.30)	17.10 (15.63–19.30)	0.003
R-LM index	0.00 (0.00–13.65)	0.00 (0.00–0.00)	<0.001
R-LM-arousal index	0.00 (0.00–1.28)	0.00 (0.00–0.00)	<0.001

Data are presented as mean ± standard deviation (SD) for normally distributed variables, and median (interquartile range, IQR) for non-normally distributed variables. Sleep efficiency (%) = total sleep time/time in bed × 100%. Arousal index (events/h) = number of arousals/total sleep time (hours). Abbreviations: REM, rapid eye movement; TST, Total sleep time; R-LM, Limb movements during REM sleep; RBD, REM sleep behavior disorder; HCs, healthy controls.

### 3.2 Technical validation of MSIF-Net

**Figure 3:**
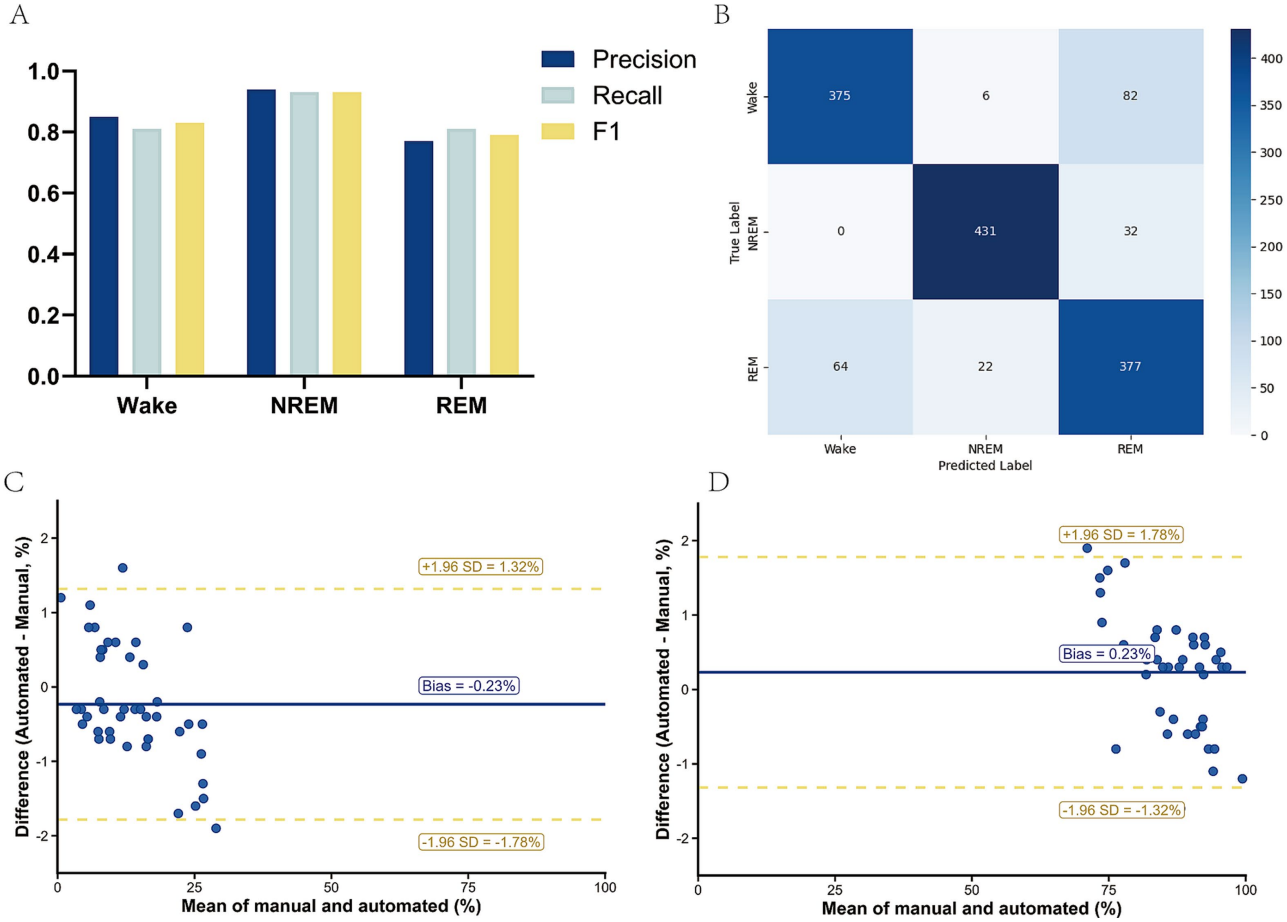
Performance of the automated sleep-staging algorithm in patients with RBD. **(A)** Stage-wise precision, recall, and F1 score for Wake, NREM, and REM, calculated on a class-balanced held-out subset used for visualization. **(B)** Confusion matrix comparing automated sleep-stage classifications with manual scoring on the same class-balanced held-out subset. **(C,D)** Bland–Altman plots comparing automated and manual estimates of whole-night stage proportions (of total sleep time, TST) for **(C)** REM% and **(D)** NREM% across participants. The solid line indicates the mean bias (automated − manual), and the dashed lines indicate the 95% limits of agreement (mean ± 1.96 SD). For REM%, the mean bias was −0.23%, with limits of agreement from −1.78 to 1.32%. For NREM%, the mean bias was 0.23%, with limits of agreement from −1.32 to 1.78%.

Table 3 summarizes the primary epoch-level classification performance based on pooled out-of-fold predictions across the patient-wise five-fold cross-validation. MSIF-Net achieved the strongest performance for NREM sleep (precision 0.95, recall 0.93, F1 = 0.94), followed by Wake (precision 0.85, recall 0.83, F1 = 0.84). REM staging remained the most challenging class (precision 0.79, recall 0.81, F1 = 0.80), with errors predominantly reflecting REM–Wake confusions. For visualization only, we additionally displayed stage-wise metrics and the confusion matrix on a class-balanced held-out subset of pooled out-of-fold epochs (N = 1,389; 463 per class) (Figures 3A,B), in order to avoid visual dominance by the majority class. Overall, these findings indicate that MSIF-Net achieved robust epoch-level discrimination in RBD, while the dominant residual errors were concentrated at the REM–Wake boundary. Nonetheless, the observed REM–Wake confusions highlight the need for further refinement to improve fine-grained epoch-level detection.

**Table 3:** Epoch-level classification performance of MSIF-Net based on pooled out-of-fold predictions in Stage 1 technical validation.

Stage	Precision	Recall	F1-score
Wake	0.85	0.83	0.84
NREM	0.95	0.93	0.94
REM	0.79	0.81	0.80

Metrics were computed by pooling held-out predictions across all five patient-wise cross-validation folds; each epoch was evaluated only when its source participant belonged to the held-out fold.

### 3.3 Clinical agreement on whole-night stage proportions

Bland–Altman analysis showed good agreement between automated and manual estimates of whole-night stage proportions in the RBD cohort (*n* = 44) (Figures 3C,D; Table 4). For REM% (of TST), the mean bias (automated − manual) was −0.23%, with 95% limits of agreement from −1.78% to 1.32%; the MAE was 0.70 percentage points. For NREM% (of TST), the mean bias was 0.23%, with 95% limits of agreement from −1.32% to 1.78%; the MAE was 0.70 percentage points. Consistent with these findings, absolute agreement between automated and manual estimates was excellent for both REM% and NREM% (ICC = 0.994, 95% CI 0.989 to 0.997). Overall, the small biases, narrow limits of agreement, and high ICC indicate that MSIF-Net provides whole-night stage-proportion estimates comparable to manual scoring.

**Table 4:** Bland–Altman agreement between automated and manual estimates of whole-night REM% and NREM% (of total sleep time) in the Stage 2 RBD cohort.

Variable	Mean bias (automated−manual), %	95% limits of agreement, %	MAE, percentage points
REM proportion (% of TST)	−0.23	−1.78 to 1.32	0.70
NREM proportion (% of TST)	0.23	−1.32 to 1.78	0.70

Agreement was assessed using Bland–Altman analysis in the Stage 2 RBD cohort. Stage proportions were expressed as percentages of total sleep time (TST). Mean bias was calculated as automated minus manual estimates, and 95% limits of agreement were defined as mean bias ± 1.96 SD of the paired differences. MAE is reported in percentage points.

### 3.4 Clinical associations between automated REM proportion and symptom scales

**Figure 4:**
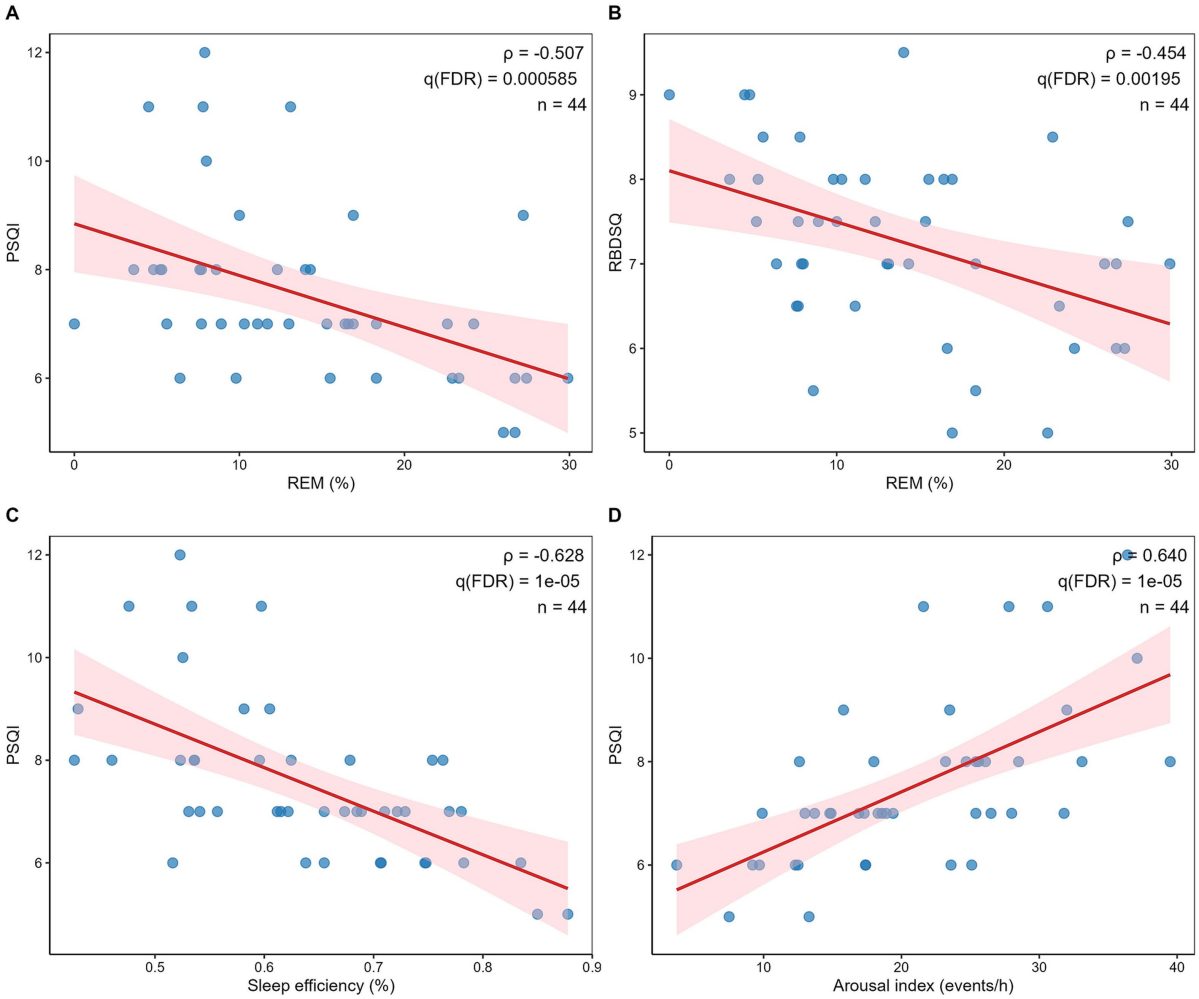
Associations between sleep parameters and clinical scales in patients with RBD (*n* = 44). **(A)** Spearman correlation between automated REM% (of TST) and PSQI (*ρ* = −0.507, *q*(FDR) = 0.000585). **(B)** Spearman correlation between automated REM% (of TST) and RBDSQ (*ρ* = −0.454, *q*(FDR) = 0.00195). **(C)** Spearman correlation between sleep efficiency and PSQI (*ρ* = −0.628, *q*(FDR) = 1 × 10^−5^). **(D)** Spearman correlation between arousal index and PSQI (*ρ* = 0.640, *q*(FDR) = 1 × 10^−5^). Red line indicates the fitted linear trend for visualization, and the shaded area denotes the 95% confidence band. All *q* values were obtained using FDR correction across the correlation tests. Abbreviations: PSQI, Pittsburgh Sleep Quality Index; ESS, Epworth Sleepiness Scale; RBDSQ, REM Sleep Behavior Disorder Screening Questionnaire; TST, total sleep time.

Automated REM% estimates derived from MSIF-Net were significantly associated with symptom burden in the RBD cohort. REM% showed a moderate negative correlation with PSQI (Spearman’s *ρ* = −0.507, *q* (FDR) < 0.001) (Figure 4A) and RBDSQ (*ρ* = −0.454, *q* (FDR) = 0.002) (Figure 4B), suggesting that lower REM% was associated with poorer subjective sleep quality and more severe RBD symptoms. No significant association was observed between REM% and ESS (*ρ* = −0.102, *q*(FDR) = 0.667). Together, these correlations suggest that MSIF-Net–derived REM% captures clinically relevant variation in sleep architecture in RBD and may provide a scalable marker linked to subjective sleep quality and symptom severity.

### 3.5 Associations of conventional PSG parameters with clinical scales

To contextualize these algorithm-derived findings, we examined associations between conventional PSG parameters and clinical scales. Sleep efficiency was strongly and inversely correlated with PSQI (*ρ* = −0.628, *q*(FDR) < 0.001) (Figure 4C), whereas the arousal index showed a strong positive correlation with PSQI (*ρ* = 0.640, *q*(FDR) < 0.001) (Figure 4D). In contrast, neither sleep efficiency nor arousal index was significantly associated with ESS or RBDSQ after FDR correction, and sleep latency was not significantly correlated with any clinical scale (all *q*(FDR) > 0.5). Collectively, conventional PSG indices primarily reflected perceived sleep quality, while automated REM% provided complementary information related to RBD symptom severity.

### 3.6 Multiple regression analysis

**Figure 5:**
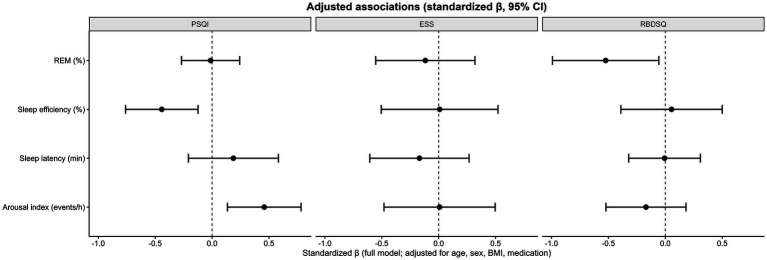
Adjusted associations between sleep parameters and clinical symptom scales in patients with RBD. Multivariable linear regression models examining the independent associations of automated REM%, sleep efficiency, sleep latency, and arousal index with PSQI, ESS, and RBDSQ scores. Standardized regression coefficients (β) and 95% confidence intervals are shown. All models were adjusted for age, sex, BMI, and medication use. The vertical dashed line indicates β = 0. REM% was independently associated with RBDSQ but not with PSQI or ESS, whereas sleep efficiency and arousal index were independently associated with PSQI.

Multiple multivariable linear regression models were constructed to evaluate the independent associations between sleep parameters and clinical symptom scales after adjustment for age, sex, BMI, and medication use (Figure 5). For subjective sleep quality (PSQI), lower sleep efficiency and higher arousal index were independently associated with higher PSQI scores, indicating poorer perceived sleep quality. In contrast, automated REM% and sleep latency did not show independent associations with PSQI after multivariable adjustment. Together, these findings suggest that conventional markers of sleep continuity and fragmentation remain the primary determinants of subjective sleep quality in patients with RBD. For daytime sleepiness (ESS), none of the examined sleep parameters—including automated REM%, sleep efficiency, sleep latency, or arousal index—demonstrated significant independent associations, consistent with the weak correlations observed in univariable analyses. In contrast, automated REM sleep proportion remained independently associated with RBD symptom severity (RBDSQ), with lower REM percentage associated with higher RBDSQ scores after adjustment for demographic factors, medication use, and conventional PSG parameters. Other sleep continuity measures did not show significant independent associations with RBDSQ. Overall, these regression analyses indicate a dissociation between sleep continuity–related outcomes and RBD-specific symptom severity: while subjective sleep quality is primarily driven by sleep efficiency and arousal burden, REM% derived from automated staging captures clinically relevant variance in RBD symptom severity beyond conventional PSG metrics.

## 4 Discussion

In this study, we developed and evaluated MSIF-Net, an automated EEG-based sleep-staging model tailored to patients with RBD, and examined the clinical relevance of model-derived REM estimates. Rather than proposing a new backbone architecture, our primary contribution is a pathology-oriented representation and adaptive fusion strategy designed for the atypical REM features encountered in RBD, together with explicit technical validation and clinical translation in independent RBD cohorts. MSIF-Net demonstrated robust epoch-level performance on pooled out-of-fold predictions, with class-wise F1 scores of 0.84 for Wake, 0.94 for NREM, and 0.80 for REM, despite residual difficulty at the REM–Wake boundary. Importantly, when applied to full-night PSG recordings from an independent clinical RBD cohort, automated estimates of whole-night REM% and NREM% showed close agreement with routine manual PSG summaries, with small mean biases and narrow limits of agreement in Bland–Altman analyses. This cohort-level validation, based on real-world laboratory PSG that inherently contains variable noise and artifacts, provides pragmatic evidence that MSIF-Net can yield stable sleep-architecture summaries under clinical conditions beyond curated epoch testing. Clinically, automated REM% correlated negatively with PSQI and RBDSQ, and remained an independent predictor of RBDSQ in adjusted models, whereas conventional continuity measures (sleep efficiency and arousal index) independently predicted PSQI. Collectively, these findings support MSIF-Net as a robust staging tool for RBD and suggest that automated, scalable estimates of REM% may provide a REM-architecture–focused summary that is informationally distinct from conventional PSG continuity indices (e.g., sleep efficiency and arousal index) and may aid clinical and research characterization of RBD.

### 4.1 MSIF-Net performance and methodological considerations

Recent RBD-oriented automated approaches are evolving along several complementary tracks. First, pathology-focused REM detection studies have shown that models performing well in general PSG cohorts may degrade in RBD, particularly in PD with RBD, underscoring the challenge posed by atypical REM physiology (34). Second, integrated frameworks such as MtRBD are beginning to combine sleep staging with RSWA detection, moving closer to clinically relevant RBD-oriented analytical pipelines (35). Third, home-based wearable studies indicate growing interest in low-burden, multi-night screening approaches (36). However, these methods primarily target movement-based screening rather than EEG-based, stage-resolved characterization of sleep architecture. Within this broader landscape, our study specifically addresses pathology-aware three-class sleep staging in clinically acquired RBD PSG and emphasizes agreement on whole-night REM/NREM architecture summaries, thereby complementing rather than replacing multimodal diagnostic and screening approaches.

More specifically, prior work consistently suggests that REM detection performance deteriorates in RBD and PD relative to control cohorts, with errors frequently driven by REM–wake confusions under atypical REM physiology (32, 34, 43). For example, a U-Sleep–based REM detector reported lower REM performance in participants with RBD than in non-RBD individuals and demonstrated prominent false-positive REM classifications during wakefulness (34). In the same study, reduced sensitivity in the RBD group was accompanied by lower specificity for light NREM stages (N1/N2), underscoring the broader challenge of stage boundary instability in pathological sleep. Several disease- and recording-related factors plausibly contribute to these error patterns (32). Loss of REM atonia and concomitant motor activity can introduce high-frequency contamination and reduce EEG contrast between REM and quiet wake, weakening canonical stage cues. In addition, RBD sleep is often fragmented, with frequent brief arousals and rapid state transitions. Within a 30-s epoch, mixed-state segments may occur, increasing label ambiguity even for expert scorers and inflating apparent disagreement (44). Medication exposure and comorbid neurodegenerative pathology may further alter REM microstructure and spectral signatures, adding inter-individual variability that challenges generalization from models trained predominantly on non-RBD datasets. These findings suggest that REM–Wake confusion in the present study should be interpreted primarily as a disease- and boundary-related error mode under pathological REM conditions, rather than as an isolated model-specific artifact.

From a methodological standpoint, the use of complementary EEG representations is supported by prior sleep-staging literature indicating that waveform-based and time–frequency–based models can both achieve strong performance while capturing different signal attributes (e.g., transient morphology versus oscillatory structure) (23, 45–47). In RBD, REM sleep is frequently atypical because loss of normal atonia and motor activity can increase contamination and blur conventional stage boundaries (48, 49). Consistent with this, quantitative EEG studies in RBD or PD have reported alterations spanning both time- and frequency-domain phenomena, including changes in sleep microstructure (e.g., spindle-related activity) and broader shifts in sleep EEG dynamics (50, 51). Together, these observations motivate a representation strategy in which (1) a time-domain stream captures transient morphology and local dynamics, (2) a time–frequency spectrogram stream encodes canonical oscillatory patterns and their disruption, and (3) handcrafted spectral descriptors provide domain-informed, clinically interpretable summaries that may be comparatively stable in modest-sized clinical datasets and have been widely used in sleep-staging models (52).

Building on this rationale, MSIF-Net was developed in response to REM–wake confusions and related stage-boundary instability in clinically acquired PSG. First, we adopted a pragmatic three-class formulation focusing on the clinically salient separation of REM from non-REM. Second, the multi-stream architecture integrates raw EEG, time–frequency spectrograms, and handcrafted features within a unified framework, and an attention-based fusion module provides adaptive feature reweighting at the epoch level. Third, the model was evaluated directly in RBD PSG rather than relying solely on predominantly non-RBD public datasets, providing a pragmatic assessment of performance under pathological REM conditions. Finally, our EEG-only design is consistent with prior work showing that automated sleep staging—including REM classification—can be performed using EEG signals alone, without requiring EMG for staging purposes, thereby supporting the feasibility of lower-burden staging workflows (53–55). However, this should not be interpreted as replacing multimodal PSG, which remains essential for comprehensive RBD evaluation and for direct assessment of RSWA-related pathophysiology. Accordingly, the present framework should not be interpreted as a diagnostic model for RBD or RSWA, but rather as an EEG-only, REM-focused staging approach intended to estimate sleep architecture under pathological REM conditions. Although strict cross-study comparison remains limited by differences in sensor montage and staging formulation, our findings suggest that EEG-only staging may still provide clinically interpretable REM/NREM architecture estimates in RBD under pathological REM conditions.

Direct head-to-head benchmarking of MSIF-Net against previously published automated sleep-staging systems was not undertaken, primarily because of fundamental differences in task formulation, input modalities, and evaluation cohorts across studies. Prior work varies substantially in montage requirements (EEG-only vs. EEG combined with EOG or EMG), staging granularity (3-class vs. 5-class or finer), epoch selection strategies, and—most critically—the populations on which models are trained and evaluated. Many widely cited architectures are developed and validated predominantly on public datasets or non-neurodegenerative cohorts, where REM physiology is relatively typical, limiting the interpretability of direct performance comparisons in patients with RBD or PD. Under such heterogeneity, apparent performance differences may reflect cohort composition or labeling conventions rather than true methodological superiority. In addition, clinical PSG recordings in RBD are intrinsically noisier than curated benchmark datasets, owing to increased motor activity during REM sleep, frequent arousals, and medication-related alterations in EEG dynamics. These factors can degrade signal quality and introduce epoch-level ambiguity even for expert manual scorers, thereby inflating apparent disagreement and complicating strict algorithmic comparisons. Our evaluation strategy therefore prioritized assessment within clinically acquired RBD PSG and aligned the evaluation targets with the aims of the study, combining epoch-level discrimination in Stage 1 with clinically interpretable whole-night REM/NREM architecture summaries in Stage 2, rather than relying solely on benchmark-style performance under more idealized conditions. Nonetheless, to clarify the relationship between the present work and previously published RBD-relevant automated sleep analysis studies, we provide a structured comparison in Supplementary Table S1.

We further acknowledge that formal ablation analyses isolating the contribution of individual representation streams and the attention-based fusion module were not performed in the present study. Given the limited sample size of currently available clinical RBD datasets and the difficulty of reliably estimating component-wise effects under high-noise conditions, we therefore emphasized end-to-end evaluation on clinical RBD PSG recordings and the clinical interpretability of model outputs, rather than exhaustive architectural tuning or component-wise optimization. Future work should incorporate systematic ablation, controlled noise perturbation, and external multicenter validation to more precisely delineate the contribution of each model component and to assess robustness across heterogeneous acquisition settings.

### 4.2 Clinical and physiological implications of altered sleep architecture in RBD

Because sleep staging is performed in 30-s epochs, automated and manual labels are expected to differ at the segment level, particularly around brief arousals and rapid state transitions that are common in RBD. However, clinical interpretation and decision-making more often rely on aggregated measures of whole-night sleep architecture than on exact epoch-by-epoch concordance. Accordingly, we evaluated whether MSIF-Net could provide clinically interpretable estimates of whole-night NREM% and REM% relative to TST by assessing agreement with routine manual PSG summaries and by examining the clinical correlates of automated REM%. By defining REM% and NREM% relative to TST rather than total recording time, we reduced conflation between sleep-stage composition and wake-related factors such as sleep latency, wake after sleep onset, terminal wakefulness, and time in bed, thereby enabling a more focused evaluation of REM/NREM architecture in RBD. Notably, despite residual epoch-level ambiguity—particularly at the REM–Wake boundary—Bland–Altman analyses demonstrated minimal mean bias and narrow limits of agreement for whole-night NREM% and REM%. These metrics operate at different levels of granularity and are not expected to align numerically: epoch-level metrics quantify local classification agreement for individual 30-s segments, whereas whole-night proportions reflect the cumulative architecture across hundreds of epochs, where boundary ambiguity and isolated misclassifications are attenuated by aggregation, especially when errors cluster near stage transitions rather than persist across stable stage periods. In this context, occasional errors—especially near REM–NREM or REM–wake transitions—are unlikely to materially change night-level stage proportions, supporting the utility of MSIF-Net for REM-focused sleep-architecture estimation in RBD rather than as a comprehensive validation of all night-level stage estimates.

Beyond technical validation, the Stage-2 clinical analyses suggest that automated REM% captures clinically meaningful variation within the RBD cohort. Specifically, MSIF-Net–derived REM% showed significant inverse associations with PSQI and RBDSQ, indicating that individuals with lower REM% tended to report poorer subjective sleep quality and greater RBD symptom burden. In multivariable models, automated REM% remained independently associated with RBDSQ after adjustment for demographic factors and relevant clinical covariates, supporting the clinical salience of REM-focused architecture estimates derived from automated staging. In contrast, REM% was not associated with ESS, suggesting that daytime sleepiness in RBD may be driven by factors beyond REM loss per se, including sleep fragmentation, medication effects, and degeneration of wake-promoting systems. The lack of a significant association with ESS further suggests that, at its current stage, automated REM% may reflect selected aspects of nocturnal sleep architecture rather than serve as a reliable surrogate for daytime functional impairment.

Manual PSG continuity metrics provided complementary context. Consistent with the subjective construct measured by PSQI, sleep efficiency and arousal index showed robust associations with PSQI, underscoring that sleep continuity and fragmentation are primary determinants of perceived sleep quality in this cohort. By contrast, these global continuity indices were not associated with ESS or RBDSQ, which is plausible given that daytime sleepiness is multifactorial and that RBDSQ reflects symptom presence and frequency rather than sleep continuity alone. Together, these findings indicate differential association patterns: sleep continuity indices (sleep efficiency and arousal index) were more closely related to global sleep quality (PSQI), whereas REM% was more closely related to RBD symptom burden (RBDSQ). This suggests that REM-architecture summaries derived automatically under pathological REM conditions may provide information that is complementary to conventional PSG continuity measures.

While we did not assess longitudinal neurodegenerative outcomes, REM-stage abnormalities are central to RBD pathophysiology and have been linked to disease trajectories in synucleinopathies in prior work (56–58). Our findings that automated REM% relates to RBDSQ are consistent with the clinical importance of REM-stage alterations in RBD, although stage proportions alone are not specific to RBD and may be influenced by comorbidities and medications. Accordingly, the present results support automated staging and clinically interpretable REM characterization in diagnosed RBD rather than prodromal detection. Future studies should integrate RBD-specific physiological markers (e.g., RSWA/RBE from EMG and EEG microstructure features) and validate predictive utility in prospective cohorts, including longitudinal outcomes relevant to neurodegenerative progression.

### 4.3 Limitations and future directions

This study has several limitations. First, model development and technical validation were performed on a limited number of RBD PSG recordings (Stage 1 cohort), and clinical translation was evaluated in a single-center cohort (*n* = 44). Although we observed consistent night-level agreement and clinically meaningful associations, broader generalizability remains uncertain. Future work should validate MSIF-Net in larger, multicenter datasets with heterogeneous acquisition conditions, including variable artifact burden, channel loss, and differences in montage and hardware. Second, MSIF-Net was intentionally formulated as a 3-class model (Wake/NREM/REM). This improves robustness under pathological REM and unstable NREM boundaries in RBD but does not support NREM substaging (N1/N2/N3) or microstructural markers (e.g., spindles, K-complexes). Accordingly, the present framework should be interpreted as a REM-focused, pathology-aware staging approach rather than a substitute for comprehensive clinical sleep architecture analysis that includes full NREM substaging. As a result, clinically relevant abnormalities in sleep depth, including fragmented or reduced deep sleep, may not be fully captured when N1, N2, and N3 are merged into a single NREM category. Extending the model to finer-grained staging—potentially via hierarchical classification or multi-task learning with microstructure detection—may improve interpretability, preserve sleep-depth information, and enable richer phenotyping. Third, the model relied exclusively on EEG input and therefore could not directly leverage EOG-defined rapid eye movements or EMG-based assessment of RSWA, both of which are central to standard REM scoring and especially important for RBD diagnosis. This design may have contributed to REM–Wake ambiguity and constrains the clinical scope of the present framework to REM-focused sleep-architecture estimation rather than RBD diagnosis. Fourth, we did not perform direct head-to-head benchmarking against matched multimodal sleep-staging systems on the same dataset. Although Supplementary Table S1 provides a structured comparison with prior published studies, direct numerical comparison remains challenging because published methods differ substantially in input modality, task formulation, staging granularity, cohort composition, and validation strategy. Meaningful benchmarking will require harmonized signal inputs, label definitions, evaluation targets, and shared clinical datasets.

Fifth, clinical translation focused on night-level stage proportions rather than additional epoch-by-epoch agreement analyses. Given strong within-subject temporal dependence across epochs and the clinical emphasis on summary sleep architecture, we prioritized whole-night NREM% and REM% (of TST) as the validation target. Nonetheless, future studies should include pre-specified epoch-level agreement analyses (e.g., boundary-specific error characterization and transition-level evaluation) and, when feasible, adjudicated labels for ambiguous epochs. Finally, correlation and regression analyses were exploratory and cross-sectional. Although we controlled for key covariates, residual confounding and multiple testing remain possible, and monotonic models may miss non-linear or threshold relationships. In addition, the clinical association analyses relied partly on subjective questionnaires, which may be influenced by recall bias and may not fully capture objective daytime functional impairment. The absence of a significant association with ESS suggests that automated REM% should not currently be interpreted as a reliable proxy for daytime sleepiness or objective daytime functional impairment in RBD. Larger samples with pre-registered hypotheses and flexible modeling approaches (e.g., generalized additive models and interaction testing) are needed, ideally alongside longitudinal follow-up to test whether algorithm-derived REM metrics track symptom trajectories, treatment response, or phenoconversion to synucleinopathies.

Looking ahead, several directions may strengthen the translational value of this work: (1) extending the framework to finer-grained staging and REM microstructure quantification (e.g., RSWA- and RBE-related metrics), (2) systematic robustness evaluation under controlled perturbations (e.g., channel dropout and additive noise) coupled with objective signal-quality indices, and (3) prospective longitudinal studies to determine whether automated REM metrics track disease progression, treatment response, or phenoconversion in neurodegenerative sleep populations. More broadly, we hope this work encourages greater attention to pathology-aware automated characterization of sleep-architecture disruption in RBD.

## 5 Conclusion

In summary, MSIF-Net demonstrated robust EEG-based three-class sleep staging performance in patients with RBD, with clinically consistent whole-night estimates of REM% and NREM% relative to TST. When applied to an independent clinical RBD cohort, automated REM% showed clinically meaningful associations with symptom scales, supporting the potential utility of REM-focused architecture summaries as a complement to conventional PSG continuity metrics. Rather than replacing manual PSG interpretation, MSIF-Net provides a scalable framework for automated sleep-architecture characterization in noisy clinical recordings, which may facilitate efficient quantification for research workflows and longitudinal monitoring. Future multicenter studies with external validation, finer-grained staging and REM microstructure quantification, and multimodal integration will be needed to strengthen generalizability and to determine prognostic value in neurodegenerative sleep populations.

## Data Availability

The PSG data supporting the findings of this study are not publicly available due to institutional and ethical restrictions, but may be obtained from the corresponding author upon reasonable request and with appropriate data use agreements.
