# Supplementary material for: Automated EEG-based sleep staging in REM sleep behavior disorder using MSIF-Net: epoch-level validation and whole-night clinical agreement
Source: Front Neurol. 2026 May 19;17:1819351. doi: 10.3389/fneur.2026.1819351 (PMC13226109; doi:10.3389/fneur.2026.1819351)
Supplement: Supplementary file 1 [file Supplementary_file_1.docx]

Supplementary Material

# Supplementary Material S1: Deep Learning Model Architecture for Sleep Stage Classification in Patients with RBD.

## Model Overview

We developed a Multi-Stream Imaging Fusion Network (MSIF-Net) to classify sleep stages (Wake, NREM, REM) in patients with RBD using EEG recordings. The model integrates three complementary branches:(1) Signal Processing Branch (1D CNN) captures temporal dynamics of raw EEG signals. (2) Spectrogram Processing Branch (2D CNN): extracts time–frequency spectro-temporal from spectrogram representations. (3) Handcrafted Feature Branch: incorporates manually extracted time- and frequency-domain features. Outputs from the three branches are fused into a unified representation via a feature-wise gating module, which adaptively weights informative features. A fully connected classifier then maps the fused features into the three sleep stage categories.

## Branch-specific Architectures

### Signal Processing Branch (1D CNN):

This branch takes 30-s EEG epochs (8 channels, 100 Hz sampling rate; 3000 time points per channel) as input. Temporal convolutional filters are applied sequentially to capture local dynamics in the EEG signal. Each convolutional layer is followed by batch normalization, ReLU activation, and max-pooling to stabilize training and progressively downsample the temporal resolution. After three convolutional blocks, adaptive global average pooling aggregates the learned representations into a compact 64-dimensional feature vector.

### Spectrogram Processing Branch (2D CNN):

For each EEG channel, time–frequency representations were computed using STFT and converted into 32×32 spectrogram images. These spectrograms, stacked across channels, were used as input to a 2D CNN. Convolutional kernels scan across both time and frequency dimensions, enabling the network to capture spectro-temporal patterns and cross-channel feature interactions. Similar to the 1D branch, each convolutional layer is followed by batch normalization, ReLU activation, and max-pooling. After three convolutional blocks, adaptive average pooling produces a 64-dimensional embedding summarizing the spectral information.

### Handcrafted Feature Branch:

Each 30-s epoch is represented by a 65-dimensional vector, including time-domain amplitude, variability statistics, frequency-domain band-limited power and spectral edge frequency (see Methods section).

### Feature Fusion and Classifier:

The fused 193-dimensional feature vector $f$ was processed by a two-layer attention MLP (193→96→193) with ReLU and sigmoid activations (37,345 parameters) to generate a 193-dimensional gating vector $\alpha\in(0,1)^{193}$. The gated representation was obtained by element-wise reweighting, $f^{*}=\alpha\odot f$, and $f^{*}$was then fed into a three-layer fully connected classifier (193→256→128→3) with batch normalization, ReLU, and dropout (0.5) to produce the final three-class output.

## Implementation Notes

(1) All convolutions use zero-padding to preserve temporal/spatial resolution before pooling.

(2) ReLU activation follows every normalization layer.

(3) Adaptive average pooling ensures fixed feature length regardless of segment duration.

(4) Dropout (0.5) was applied to fully connected layers to mitigate overfitting.

(5) The model was implemented in PyTorch (version 2.2).

# Supplementary Material S2: Handcrafted Features: Definition, Role, and Selection

## Overview and role in MSIF-Net.

To complement the learned representations from the 1D- and 2D-CNN streams, we extracted handcrafted EEG features for each 30-s epoch. The purpose of this branch was to provide (1) physiologically interpretable descriptors widely used in sleep research and clinical EEG interpretation, and (2) stable summary statistics that may help model robustness when sample size is limited and when clinical PSG data contain variable noise and artifacts. These handcrafted features were fused with CNN embeddings through the attention module.

## Candidate feature pool (80 features).

We initially computed 80 candidate descriptors across the eight channels (Fp1, Fp2, C3, C4, O1, O2, A1, A2), grouped as follows:

Time-domain statistics (32 features). Mean, standard deviation, variance, and root mean square (RMS) computed per channel. The time-domain variability measures capture epoch-level signal stability and amplitude fluctuations, which can change with arousals and movement-related contamination.

Frequency-domain band power (40 features). Absolute band-limited power in delta (0.5–4 Hz), theta (4–8 Hz), alpha (8–13 Hz), beta (13–30 Hz), and low-gamma (30–35 Hz given the 0.5–35 Hz bandpass) bands per channel. Power spectral density was estimated using Welch’s method (fs = 100 Hz; nperseg = 256; Hamming window). The band-limited power features encode canonical sleep oscillations and arousal-related high-frequency activity.

Spectral edge frequency (8 features). SEF90 per channel, defined as the frequency below which 90% of the total spectral power is contained. The SEF90 summarizes spectral shifts toward higher frequencies and provides a compact indicator of arousal-related changes in EEG.

## Derivation of the final 65-dimensional vector used in the model.

Although 80 handcrafted descriptors were computed for completeness (Table S2), a fixed 65-feature subset was predefined a priori and used as the handcrafted input to MSIF-Net to maintain a consistent dimensionality and reduce redundancy. Features included in the model are indicated in the “Used in model” column. Briefly, we retained all time-domain statistics, band-power features from the six scalp channels (Fp1, Fp2, C3, C4, O1, O2), and SEF90 from representative channels (Fp1, Fp2, C3), while excluding band-power and SEF90 features from mastoid reference channels (A1/A2).

# Supplementary Tables

## Supplementary Table 1. Detailed architecture of MSIF-Net for sleep stage classification.

| **Branch** | **Layer** | **Type** | **Input Size** | **Output Size** | **Parameters** |
| --- | --- | --- | --- | --- | --- |
| Signal Processing Branch (1D CNN) | 1 | Conv1d (in=8, out=16, kernel=5, stride=1, padding=2) | [B,8,3000] | [B,16,3000] | 656 |
|  | 2 | BN + ReLU + MaxPool1d (kernel=2, stride=2) | [B,16,3000] | [B,16,1500] | 32 |
|  | 3 | Conv1d (16→32, k=5, s=1, p=2) | [B,16,1500] | [B,32,1500] | 2,592 |
|  | 4 | BN + ReLU + MaxPool1d (2,2) | [B,32,1500] | [B,32,750] | 64 |
|  | 5 | Conv1d (32→64, k=5, s=1, p=2) | [B,32,750] | [B,64,750] | 10,304 |
|  | 6 | BN + ReLU + MaxPool1d (2,2) | [B,64,750] | [B,64,375] | 128 |
|  | 7 | AdaptiveAvgPool1d (1) + Flatten | [B,64,375] | [B,64] | 0 |
| Spectrogram Processing Branch (2D CNN) | 1 | Conv2d (in=8, out=16, k=3, s=1, p=1) | [B,8,32,32] | [B,16,32,32] | 1,168 |
|  | 2 | BN + ReLU + MaxPool2d (2,2) | [B,16,32,32] | [B,16,16,16] | 32 |
|  | 3 | Conv2d (16→32, k=3, s=1, p=1) | [B,16,16,16] | [B,32,16,16] | 4,640 |
|  | 4 | BN + ReLU + MaxPool2d (2,2) | [B,32,16,16] | [B,32,8,8] | 64 |
|  | 5 | Conv2d (32→64, k=3, s=1, p=1) | [B,32,8,8] | [B,64,8,8] | 18,496 |
|  | 6 | BN + ReLU + MaxPool2d (2,2) | [B,64,8,8] | [B,64,4,4] | 128 |
|  | 7 | AdaptiveAvgPool2d (1) + Flatten | [B,64,4,4] | [B,64] | 0 |
| Handcrafted Feature Branch | – | Pre-extracted | 65-dim | – | – |
| Fusion & Classifier | 1 | Attention MLP | 193 →96→ 193 | 193-dim | 37,345 |
|  | 2 | Linear + BN + ReLU + Dropout | 193 → 256 | – | 50,688 |
|  | 3 | Linear + BN + ReLU + Dropout | 256 → 128 | – | 33,152 |
|  | 4 | Linear | 128 → 3 | – | 387 |
| Total（Attention + Classifier） | – | – | – | – | 159,876 |

## Supplementary Table 2. Fold-wise epoch-level classification performance on held-out test folds across five patient-wise cross-validation iterations

| **Fold** | **Wake Precision** | **Wake Recall** | **Wake F1-score** | **NREM Precision** | **NREM Recall** | **NREM F1-score** | **REM Precision** | **REM Recall** | **REM F1-score** |
| --- | --- | --- | --- | --- | --- | --- | --- | --- | --- |
| Fold 1 | 0.85 | 0.81 | 0.83 | 0.95 | 0.93 | 0.94 | 0.76 | 0.80 | 0.78 |
| Fold 2 | 0.87 | 0.84 | 0.85 | 0.94 | 0.93 | 0.93 | 0.78 | 0.81 | 0.80 |
| Fold 3 | 0.85 | 0.82 | 0.84 | 0.95 | 0.93 | 0.94 | 0.80 | 0.80 | 0.80 |
| Fold 4 | 0.86 | 0.84 | 0.85 | 0.93 | 0.92 | 0.93 | 0.79 | 0.82 | 0.80 |
| Fold 5 | 0.84 | 0.84 | 0.84 | 0.94 | 0.93 | 0.93 | 0.79 | 0.81 | 0.80 |
| Mean across folds | 0.85 | 0.83 | 0.84 | 0.94 | 0.93 | 0.93 | 0.78 | 0.81 | 0.80 |

**Note:** Performance metrics were calculated on the held-out test fold in each patient-wise cross-validation iteration. The “Mean across folds” row represents the arithmetic mean of the fold-wise metrics and is provided to illustrate fold-to-fold stability. Because the primary estimates in Table 3 were computed from pooled out-of-fold predictions across all folds, they are not expected to be numerically identical to the arithmetic means shown here.
