# Supplementary material for: Automated EEG-based sleep staging in REM sleep behavior disorder using MSIF-Net: epoch-level validation and whole-night clinical agreement
Source: Front Neurol. 2026 May 19;17:1819351. doi: 10.3389/fneur.2026.1819351 (PMC13226109; doi:10.3389/fneur.2026.1819351)
Supplement: Supplementary file 2 [file Table_1.docx]

**Supplementary Table S1. Positioning of the present study relative to published automated sleep staging and REM-related studies relevant to RBD**

| **Study** | **Population/cohort** | **Input modality** | **Task formulation** | **Output granularity** | **Validation setting** | **Main finding relevant to RBD** | **Distinctive contribution and remaining gap relative to the present study** |
| --- | --- | --- | --- | --- | --- | --- | --- |
| Cooray et al., 2019 | 53 patients with RBD and 53 age-matched healthy controls; RBD cohort combined from CAP and John Radcliffe datasets; controls from MASS | Limited PSG montage: 1 EEG, 1 EOG, and chin EMG | Two-stage fully automated pipeline: 5-stage sleep staging followed by subject-level RBD detection using RSWA-related metrics, sleep-architecture features, and EMG fractal features | Epoch-level 5-class sleep staging; subject-level binary RBD classification | 10-fold cross-validation across pooled multi-dataset recordings; performance evaluated under both manual and automated staging | Automated sleep staging achieved Cohen’s κ = 0.62 overall, with lower agreement in RBD than in healthy controls (κ = 0.54 vs 0.73). REM sensitivity was markedly reduced in RBD (0.45 ± 0.30), and REM epochs were frequently misclassified as wake. Despite this, subject-level RBD detection remained strong (96% accuracy with manual staging; 92% with automated staging). | A clinically important RBD-focused comparator showing that sleep-architecture information can support downstream RBD detection. However, its primary endpoint is subject-level RBD identification after staging, not direct validation of clinically interpretable sleep-stage summaries in an independent RBD cohort. It is therefore best viewed as a clinically adjacent pipeline rather than a task-matched comparator to the present EEG-only staging study. |
| Jung et al., 2025 | Multicenter real-world PSG cohort from 5 tertiary hospitals; 310 datasets total, including RBD (n = 200) and non-RBD (n = 110), further subdivided into PD with RBD (n = 76), PD without RBD (n = 46), iRBD (n = 124), and healthy controls (n = 64) | EEG + EOG only; EMG deliberately excluded | Automated REM sleep detection using a pretrained U-Sleep-based deep-learning model; focused on REM-versus-other-stage discrimination rather than full sleep staging or RBD diagnosis | Epoch-level REM detection | Multicenter retrospective validation with expert manual scoring as reference; 5-fold cross-validation repeated for ROC visualization; heterogeneous real-world PSG from 5 hospitals | Overall REM detection was good (AUC 0.90 ± 0.14), but performance declined significantly in RBD versus non-RBD (AUC 0.88 ± 0.13 vs 0.93 ± 0.14; sensitivity 58.2% vs 79.0%). Performance was worst in PD with RBD (AUC 0.86 ± 0.02). False positives in RBD were predominantly wake-related, underscoring REM–wake ambiguity under REM sleep without atonia. | Highly relevant because it directly shows that a modern REM detector degrades in disease-relevant RBD cohorts, especially PD-RBD. However, it addresses REM detection only, not full multi-stage sleep staging, whole-night architecture agreement, or subject-level clinical translation. It therefore supports the specific difficulty of REM scoring in RBD, but not the broader clinical question addressed here. |
| van der Aar et al., 2025 | 36 patients with RBD and 36 age- and sex-matched patients with mild-to-moderate OSA from the SOMNIA database; RBD group included both isolated and secondary RBD | Two automated sleep-staging paradigms: ExG-based ASSC using EEG/EOG/chin EMG from PSG, and HRVm-based ASSC using wrist-derived photoplethysmography plus accelerometry | Comparative analysis of automated sleep staging in RBD, with emphasis on categorical hypnograms and probability-based hypnodensity representations rather than direct RBD diagnosis | 4-stage sleep staging (Wake / N1+N2 / N3 / REM), plus probability-distribution measures such as ambiguity and transition continuity | Case-control comparison within a single database using manual scoring as reference; matched RBD vs OSA design | Automated staging was worse in RBD across both ExG-based and HRVm-based models (ExG κ = 0.74 vs 0.80; HRVm κ = 0.50 vs 0.63). Lower performance concentrated in N1+N2 and REM, and REM was often misclassified as N1+N2. Hypnodensity analysis revealed greater ambiguity and more continuous transition profiles in RBD, suggesting that reduced agreement reflects less distinct stage boundaries rather than sleep instability alone. | A strong mechanistic and contextual comparator because it explains why automated staging is intrinsically harder in RBD. However, it is primarily a sleep-structure characterization study, not an end-to-end clinical staging framework with independent clinical-translation evaluation. It complements, rather than replaces, the present study’s focus on pathology-aware EEG-only staging and whole-night architecture agreement. |
| Chen et al., 2025 (MtRBD) | Clinical iRBD cohort from Shanghai Changzheng Hospital; 30 patients over 59 nights (CZ-RBD dataset) | Multichannel PSG including EEG, EOG, chin EMG, and bilateral tibialis anterior EMG | Joint multi-task learning for simultaneous 5-stage sleep staging and RSWA detection, explicitly modeling the physiological relationship between the two tasks | Epoch-level 5-class sleep staging and epoch-level 3-class RSWA-related output (Non-REM / Normal REM / Abnormal REM with RSWA) | Leave-one-subject-out cross-validation on a single-center clinical iRBD dataset | One of the few studies to jointly optimize sleep staging and RSWA detection rather than treating them as sequential steps. MtRBD achieved 83.0% sleep-staging accuracy and κ = 0.756, with REM F1 = 0.816; for RSWA detection, accuracy was 93.6% with macro-F1 = 0.824 and F1 = 0.705 for abnormal REM with RSWA. | Methodologically close and highly relevant, because it jointly addresses sleep staging and an RBD-critical pathological event within a unified framework. However, it is a single-center, multimodal PSG-based multitask model that explicitly integrates EEG, EOG, and EMG signals to jointly perform sleep staging and RSWA detection, rather than EEG-only staging under constrained input conditions. The present study therefore occupies a different niche: lower-modality-burden, pathology-aware EEG-only staging with independent clinical translation of whole-night REM/NREM architecture summaries. |
| Tzfoni et al., 2026 | 73 participants: 15 PSG-confirmed iRBD and 58 controls; all underwent one laboratory vPSG night followed by up to 6 nights of home monitoring | Single lower-back wearable inertial sensor (lumbar-mounted IMU); mobility-derived nocturnal features only | Home-based machine-learning screening for subject-level iRBD detection from multi-night nocturnal mobility patterns | Subject-level binary classification (iRBD vs non-iRBD), derived from multi-night home data | Proof-of-concept study with vPSG-confirmed reference diagnosis; subject-level leave-one-out cross-validation; multi-night home validation | Demonstrated that a single lumbar wearable can detect nocturnal motor patterns associated with iRBD with high sensitivity but only moderate specificity. In the best home-setting model, sensitivity was 93.33%, specificity 72.41%, accuracy 76.71%, balanced accuracy 82.87%, and F1-score 62.22%. Performance improved with additional nights and plateaued at ~5 nights, supporting multi-night home screening as a staged strategy. | Clinically relevant but not task-matched. It does not perform sleep staging, REM detection, or RSWA quantification, but instead addresses home-based subject-level screening using wearable movement features. Its value is complementary: it supports low-burden pre-screening and cohort enrichment, whereas the present study addresses PSG-based, stage-resolved REM/NREM architecture quantification in clinically acquired RBD recordings. |
| Present study (MSIF-Net) | Two-stage, two-cohort design in clinically acquired RBD PSG: Stage 1 technical validation in 17 patients with RBD; Stage 2 independent clinical translation in 44 patients with RBD; 44 age- and sex-matched healthy controls used only for group-level PSG/clinical comparisons | EEG-only PSG montage using 8 routinely available EEG channels (Fp1, Fp2, C3, C4, O1, O2, A1, A2) | Pathology-aware automated sleep staging in RBD, focusing on REM-relevant architecture under atypical REM physiology; Stage 1 epoch-level Wake/NREM/REM classification followed by Stage 2 whole-night clinical translation | Epoch-level 3-class sleep staging; participant-level whole-night REM% and NREM% (of TST) | Stage 1: patient-wise 5-fold cross-validation with pooled out-of-fold evaluation; Stage 2: independent, non-overlapping RBD cohort for automated–manual agreement and symptom-association analyses | On pooled out-of-fold predictions, F1 scores were 0.84 for Wake, 0.94 for NREM, and 0.80 for REM, with residual errors concentrated at the REM–Wake boundary. Despite this epoch-level ambiguity, whole-night agreement with manual summaries was close (REM bias −0.23%, NREM bias 0.23%; ICC 0.994), and automated REM% correlated with PSQI and RBDSQ. | The distinctive contribution of the present study is not raw benchmark-style numerical superiority across heterogeneous settings, but clinical/methodological niche superiority: it specifically demonstrates that EEG-only, pathology-aware staging in real-world RBD PSG can yield clinically interpretable whole-night REM/NREM architecture summaries, validated in an independent clinical cohort. Unlike prior work centered on subject-level RBD detection, REM-only detectors, multitask RSWA pipelines, or wearable screening, this study provides a lower-burden yet clinically grounded bridge between epoch-level automated staging and participant-level sleep-architecture interpretation in RBD. |

**Note.** This table is intended to position the present study relative to prior RBD-relevant automated sleep staging and REM-related studies. Direct numerical comparison across studies should be interpreted cautiously because the studies differ substantially in cohort composition, input modality, task formulation, staging granularity, and validation design. Accordingly, the comparison is used here to clarify the clinical and methodological niche of the present study rather than to claim raw performance superiority across non-comparable settings.
